# One-Dimensional Oxide Nanostructures as Gas-Sensing Materials: Review and Issues

**DOI:** 10.3390/s100404083

**Published:** 2010-04-22

**Authors:** Kyoung Jin Choi, Ho Won Jang

**Affiliations:** 1 Nano-Materials Center, Korea Institute of Science and Technology, Seoul, 130-650, Korea; 2 Electronic Materials Center, Korea Institute of Science and Technology, Seoul, 130-650, Korea; E-Mail: hwjang@kist.re.kr

**Keywords:** 1-dimensional nanostructures, gas sensors, long-term stability, gas selectivity, electronic-nose, room-temperature operation

## Abstract

In this article, we review gas sensor application of one-dimensional (1D) metal-oxide nanostructures with major emphases on the types of device structure and issues for realizing practical sensors. One of the most important steps in fabricating 1D-nanostructure devices is manipulation and making electrical contacts of the nanostructures. Gas sensors based on individual 1D nanostructure, which were usually fabricated using electron-beam lithography, have been a platform technology for fundamental research. Recently, gas sensors with practical applicability were proposed, which were fabricated with an array of 1D nanostructures using scalable micro-fabrication tools. In the second part of the paper, some critical issues are pointed out including long-term stability, gas selectivity, and room-temperature operation of 1D-nanostructure-based metal-oxide gas sensors.

## 1. Introduction

In 1962, Seiyama *et al*. discovered that the electrical conductivity of ZnO could be dramatically changed by the presence of reactive gases in the air [1]. Since then, there have been tremendous reports on the applications of semiconducting metal oxides as gas sensors due to their small dimensions, low cost, and high compatibility with microelectronic processing. Recently, one-dimensional (1D) semiconductor nanostructures including nanowires, nanotubes, and nanobelts have attracted considerable interest for their potential as building blocks for fabricating various nanodevices. Due to the high surface-to-volume ratios and high crystallinity of these 1D nanostructures, their major application was first made on the chemical/gas sensors.

**Figure 1.**
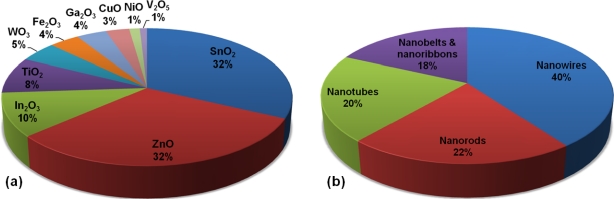
(a) Top 10 materials and (b) element forms of 1D metal oxide nanostructures used for gas sensor applications in publications since 2002. The publication search was performed using the Science Citation Index Expanded database of Web of Science provided by Thomson Reuters. For each material type, all possible keywords from combinations of gas sensor and 1D nanostructures (nanowire, nanorod, nanotube, nanobelt and nanoribbon) were used for the search.

SnO_2_ is the most widely studied material among all the oxides used for gas sensor applications. Forty-two percent of publications in last three decades focused on SnO_2_, along with ZnO (16%), TiO_2_ (13%), WO_3_ (9%), and In_2_O_3_ (7%). These oxide materials are then accompanied by Fe_2_O_3_, CuO, NiO, Ga_2_O_3_, and V_2_O_5_ in sequence. The predominance of SnO_2_ is due to the fact that the material is sensitive to all the gaseous species. For 1D metal oxide nanostructures used for gas sensor applications, the relative comparison of the top 10 oxides in the publications since 2002 is shown in Figure 1(a). Both SnO_2_ and ZnO are the most widely studied materials at 32%. In_2_O_3_ is at 10%, TiO_2_ at 8%, and WO_3_ at 5%, followed by Fe_2_O_3_, Ga_2_O_3_, CuO, NiO, and V_2_O_5_ in sequence. It is believed that the easy synthesis of high-quality and single-crystalline 1D ZnO nanostructures has led to the intensive studies in gas sensors based on 1D ZnO nanostructures. The synthesis of 1D nanostructures based on TiO_2_ and WO_3_ has however been reported to be hard compared to other oxides. Figure 1(b) shows a pie chart for element forms of 1D metal oxide nanostructures used for gas sensor applications. It is clear that nanowires are the most widely investigated form at 40%, followed by nanorods, nanotubes, and nanobelts and nanoribbons at ∼20%. The dominant materials for each form are ZnO and SnO_2_ nanowires, ZnO nanorods, SnO_2_-based nanotubes, and SnO_2_ nanobelts and nanoribbons.

In this review, gas sensors based on 1D metal-oxide nanostructures were reviewed comprehensively with major emphases on the types of device structure and issues. While gas sensors based on individual 1D nanostructures were successfully fabricated for fundamental research, devices with practical applicability were fabricated with an array of 1D nanostructures using scalable micro-fabrication tools. also In addition, some critical issues are pointed out including long-term stability, gas selectivity, and room-temperature operation of 1D-nanostructure-based metal-oxide gas sensors.

## 2. Types of Gas-Sensor Structure Based Upon 1D Oxide Nanostructures

### 2.1. Single 1D Nanostructure Gas Sensors

Law *et al.* [2] have found that individual single-crystalline SnO_2_ nanoribbons have strong photoconducting response and thus detect ppm-level NO_2_ at room temperature by illuminating the nanoribbons with UV light of energy near the SnO_2_ bandgap (*E_g_* = 3.6 eV at 300 K). Photogenerated holes recombine with trapped electrons at the surface, desorbing NO_2_ and other electron-trapping species: h^+^ + NO_2_^−^_(ads)_ → NO_2(gas)_. The space charge layer thins, and the nanoribbon conductivity rises. Ambient NO_2_ levels are tracked by monitoring changes in conductance in the illuminated state. The larger and faster response of individual nanoribbon sensors with 365 nm illumination than that with 254 nm illumination suggested that the presence of surface states plays a role in the photochemical adsorption-desorption behavior at room temperature.

Wang and co-workers demonstrated the gas sensing ability of field-effect transistors (FETs) based on a single SnO_2_ nanobelt [3]. SnO_2_ nanobelts were doped with surface oxygen vacancies by annealing in an oxygen-deficient atmosphere. Then the source-drain current of SnO_2_ nanobelt FETs could respond and recover with exposure and removal of oxygen in ambient nitrogen at 200 °C. Later, they improved the device performance of the SnO_2_ nanobelt FETs [4]. Low-resistance RuO_2_/Au Ohmic contacts on the SnO_2_ nanobelts led to high-quality n-channel depletion mode FETs with well-defined linear and saturation regimes, large on current, and on/off ratio as high as 10^7^. The FET characteristics show a significant modification upon exposure to 0.2% H_2_. The channel conductance in the linear regime increases by around 17% at all gate voltages. The hydrogen reacts with and removes the oxygen adsorbed on the metal oxide surface and thus increases the electron concentration and the conductance of the nanobelt channel [5]. Qian *et al.* [6] reported a CO sensor based on an individual Au-decorated SnO_2_ nanobelt.

Wang and co-workers presented a high sensitivity humidity sensor based on a single SnO_2_ nanowire [7]. The SnO_2_ nanowire based sensor had a fast and sensitive response to relative humidity in air from a wide range of environments at room temperature. In addition, it had relatively good reproducibility, and its linear response to 30–90% RH makes it easy to calibrate. The sensitivity of the single SnO_2_ nanowire based sensors to CO, CH_4_ and H_2_S gases at 250 °C was improved by 50–100% through surface functionalization with ZnO or NiO nanoparticles [8]. The heterojunction between the surface coating layers and SnO_2_ (*i.e*., n-n junction for ZnO-SnO_2_ and p-n junction for NiO-SnO_2_) and the corresponding coupling effect of the two sensing materials played a critical role in controlling device sensitivity. Besides heterojunctions, many other factors such as the size and crystalline state of surface additives and the concentration change of structure defects in the nanowires might bring a pronounced influence on the gas sensing performance of the SnO_2_ nanowire based device. Thus, it was difficult to use a uniform model to completely elucidate the nature of the surface additives. Despite this, it was clear that surface functionalization is a good strategy to improve the sensitivity and selectivity of the SnO_2_-based nanosensor. Kumar *et al.* [9] reported highly sensitive H_2_S sensors based on homogeneously Cu-doped SnO_2_ single nanowires. By Cu doping, the sensitivity of SnO_2_ single nanowire sensors could be increased by up to 10^5^.

**Figure 2.**
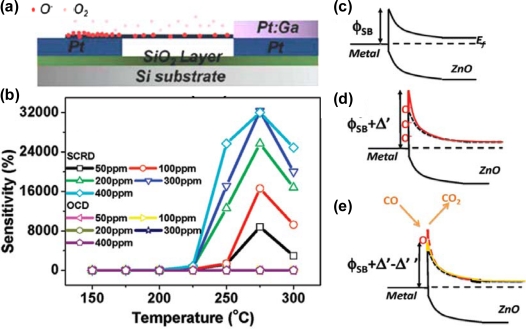
(a) A schematic of the SCD based on a single ZnO nanowire at O_2_ adsorption. (b) Sensitivity *versus* system temperature for CO sensing at a response time of 1 h as a function of the CO concentration at 275 °C. Results collected from the OCD and SCD at reverse bias (SCRD) are compared. (c–e) Schematics showing the response of Schottky barrier height in response to variations in (c) N_2_, (d) O_2_, and (e) CO atmospheres (Reprinted from reference [10] with permission from American Chemical Society).

Recently, Wang and co-workers reported gigantic enhancement of sensitivity in a single ZnO (*E_g_* = 3.37 eV at 300 K) nanowire based gas sensor with asymmetric Schottky contact [10]. The device was composed of a single ZnO nanowire mounted on Pt electrodes with one end in Pt:Ga/ZnO Ohmic contact and the other end in Pt/ZnO Schottky contact (Figure 2a). An ultrahigh sensitivity of 32000% was achieved using the Schottky contacted device (SCD) operated in reverse bias mode at 275 °C for detection of 400 ppm CO mixed with dry air, which was four orders of magnitude higher than that obtained using an Ohmic contact device (OCD) under the same conditions (Figure 2b). The local Schottky barrier height of the small contact area between the nanowire was tuned through the responsive variation of the surface chemisorbed gases at the junction area (Figures 2c–2e), which serves as a “gate” for controlling the transport of charge carriers [11,12]. In addition, the response time and reset time were shortened by a factor of seven. Liao *et al*. [13] showed that the sensitivity of gas sensor based on a single ZnO nanowire to H_2_S in air at room temperature could be modulated and enhanced by He^+^ irradiation at an appropriate dose. Choi *et al*. [14] have developed a new smart ZnO nanowire gas sensor based on the commercially available 0.35 μm complementary metal–oxide–semiconductor (CMOS) process to improve the sensing performance with better resolution and to evaluate the reliability of the single ZnO nanowire gas sensor.

**Figure 3.**
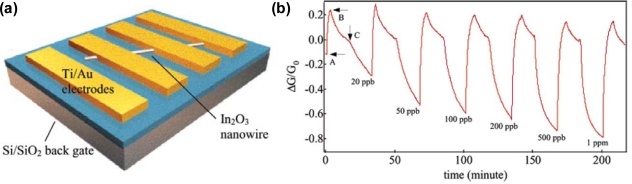
(a) A schematic of a single In_2_O_3_ nanowire sensor, where Ti/Au electrodes are deposited on nanowire-decorated Si/SiO_2_ substrate. (b) Sensing response of a single nanowire device to NO_2_ diluted in air. The normalized conductance change (ΔG/G_0_) is plotted as a function of time with the nanowire sensor exposed to NO_2_ of various concentrations. Recovery was made by UV light (254 nm) desorption of NO_2_. At point A, the first cycle was taken with UV illumination. The nanowire conductance kept rising until the UV light was turned off at point B. 20 ppb NO_2_ was introduced to the airflow at point C (Reprinted from reference [16] with permission from American Chemical Society).

Zhou and co-workers reported ultrasensitive single In_2_O_3_ nanowire sensors for NO_2_ and NH_3_ at room temperature [15]. The devices exhibited far superior performance compared to previously reported results. For instance, the devices exhibited sensitivities (defined as the resistance after exposure divided by the resistance before exposure) of 10^6^ for NO_2_ and 10^5^ for NH_3_, which are four or five orders of magnitude better than results obtained with thin-film based sensors. Response times (defined as time duration for resistance change by one order of magnitude) as short as 5 s for 100 ppm NO_2_ and 10 s for 1% NH_3_ have also been achieved. The lowest detectable gas concentrations were 0.5 ppm for NO_2_ and 0.02% for NH_3_. In addition, UV illumination of our devices can dramatically enhance the surface molecular desorption kinetics and thus lead to substantially reduced recovery time. They have further developed the device performance of the single In_2_O_3_ nanowire sensors [16], demonstrating a detection limit of NO_2_ at ∼20 ppb, which is the lowest detectable concentration ever achieved with all types of metal oxide nanowire sensors and all conventional solid-state NO_2_ sensors working at room temperature (Figure 3). Recently, Zeng *et al.* [17] demonstrated a highly sensitive and selective H_2_S nanosensor by using a single In_2_O_3_ nanowire transistor. The nanosensor worked at room temperature without UV-assisted desorption and exhibited a detection limit of 1 ppm for H_2_S. The response and recovery are both very fast at ∼50 s. Moreover, the nanosensor demonstrates an extremely weak response to NH_3_ and total insensitivity to CO, which is highly promising for practical application for detecting low concentration of H_2_S.

Moskovits and co-workers have intensively studied the electron-transport properties of single SnO_2_ nanowires configured as FETs over a wide temperature range in various atmospheres comprised of mixtures of N_2_/O_2_/CO [18–20]. Owing to their large surface-to-volume ratios, the bulk electronic properties of the nanowires were found to be controlled almost entirely by the chemical processes taking place at their surface, which could in turn be modified by controlling the gate potential. Thus, the rate and extent of oxygen ionosorption and the resulting rate and extent of catalytic CO oxidation reaction on the nanowire’s surface could be controlled and even entirely halted by applying a negative enough gate potential, presenting the prospect of tuning catalysis or other surface reactions entirely through electronic means [20].

Moskovits and co-workers have shown enhanced gas sensing of single SnO_2_ nanowires configured as resistive elements by surface decoration with metal nanoparticles such as Pd [21] and Ag [22]. For Pd-decorated SnO_2_ nanowires, the 500–1000% improvement in sensitivity toward oxygen and hydrogen was attributed to the enhanced catalytic dissociation of the molecular adsorbate on the Pd nanoparticle surfaces and the subsequent diffusion of the resultant atomic species to the oxide surface (spillover effect). For Ag-decorated SnO_2_ nanowires, the significant improvement in sensitivity toward ethylene was due to the modification of the Schottky junction formed between the Ag particles and the tin oxide resulting from the surface chemical processes involving ethylene and oxygen occurring exclusively on the silver nanoparticles’ surface (electronic effect).

**Figure 4.**
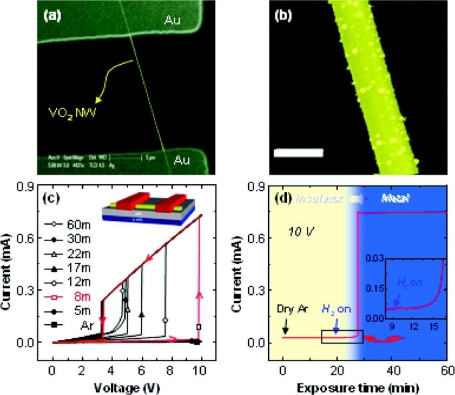
(a) Scanning electron microscopy (SEM) image of an individual VO_2_ nanowire device configured with appropriate Ohmic contacts for electrical measurements in a gaseous atmosphere. (b) SEM image of a Pd-decorated VO_2_ nanowire. The Pd particles, 5–22 nm in diameter, are noncontinuous and cover the surface of the nanowire uniformly (scale bar, 200 nm). (c) *I–V* curves obtained at 50 °C for Pd-decorated VO_2_ nanowire after various exposure times to hydrogen gas (5 sccm), added to the background argon stream (10 sccm). (d) The change in current for a Pd-decorated VO_2_ nanowire biased at 10 V as a function of time of exposure to hydrogen gas. Initially the current increases gradually with hydrogen exposure time and then at ∼7 min increases dramatically by ∼3 orders of magnitude (5 × 10^−6^ A → 6 × 10^−3^ A) in the absence of the series resistor (Reprinted from reference [24] with permission from American Chemical Society).

Recently, Stelecov *et al.* [23] have demonstrated gas sensors based on single VO_2_ nanowires, where the pressure dependent onset of metal-insulator transition in single crystal suspended VO_2_ nanowires was used as a sensor signal. Moskovits and co-workers have reported that exceptionally sensitive hydrogen sensors were produced using Pd-nanoparticle-decorated, single VO_2_ nanowires [24]. The high sensitivity arose from the large downward shift in the insulator to metal transition temperature following the adsorption on and incorporation of atomic hydrogen, produced by dissociative chemisorption on Pd, in the VO_2_, producing ∼1000-fold current increases (Figure 4).

Wang and co-workers have studied oxygen sensing properties of room-temperature single nanowire gas sensors based on various oxides such as ZnO [25], *β*-Ga_2_O_3_ [26], and ZnSnO_3_ [27]. Single *β*-Ga_2_O_3_ (*E_g_* = 4.9 eV at 300 K) nanowires exhibited a very fast oxygen response time of ∼1 s in 254 nm UV illumination [26], providing a route for realizing room-temperature fast-response oxygen sensors. *β*-Ga_2_O_3_ has very low carrier density and the oxygen sensing only appeared under the UV illumination. This is different from semiconducting nanowires including SnO_2_, In_2_O_3_, and ZnO, which initially have oxygen sensing properties due to the high carrier density without UV illumination, have long response time of several minutes under the UV illumination. Extremely high oxygen sensitivity about six orders of magnitude was realized from single ZnSnO_3_ nanowires with abundant grain boundaries [27]. Such a drastic sensing was ascribed to grain boundary barrier modulation, demonstrate a promising approach to realize miniaturized and highly sensitive oxygen sensors.

**Figure 5.**
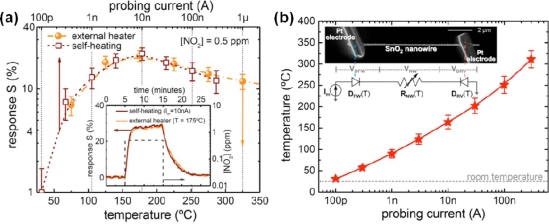
(a) Response of SnO_2_ nanowires operated in self-heating mode and with external microheater. (b) Estimated temperature of the devices at different *I_m_* (*r_nw_* = 35 nm). The inset is a SEM image of a SnO_2_ nanowire connected to two Pt microelectrodes fabricated with focused ion beam. The equivalent circuit of this structure corresponds to two back-to-back diodes (D_FW_ and D_RV_) in series with the nanowire resistance (R_NW_). These three components dissipate electrical power and contribute to the self-heating of the device (Reprinted from reference [29] with permission from American Institute of Physics).

**Figure 6.**
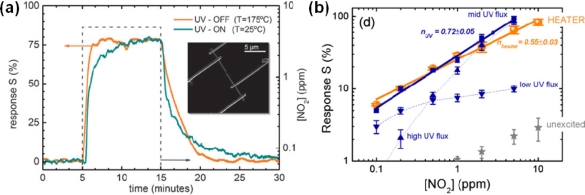
(a) Comparison of the response of a SnO_2_ nanowire, operated at T = 175 °C in dark conditions and at room temperature (T = 25 °C) under UV illumination (*E_ph_* = 3.67 ± 0.05 eV, *Φ_ph_* = 30 × 10^22^ ph/m^2^s) to a pulse of 5 ppm [30]. (b) Comparison of the sensor response when operated with conventional heating (*T* = 175 °C) and UV illumination. The selection of the appropriate photon flux leads to sensor performances comparable to those of conventional heated sensors (Reprinted from reference [31] with permission from American Institute of Physics).

Morante and co-workers have provided a systematic study on effects of contact resistances and the nanowire diameter size on the CO and humidity measurements using a single SnO_2_ nanowire [28]. Controlled AC impedance measurements revealed that the single SnO_2_ nanowire sensor had CO detection threshold smaller than 5 ppm and measurement instability lower than 4% at 295 °C. They we have demonstrated ultralow power consumption of self-heated single SnO_2_ nanowire gas sensors [29]. For instance, the response of the sensors to 0.5 ppm NO_2_ without heater (*I_m_* = 10 nA) was the absolute equivalent to that with a heater (*T* = 175 °C) (Figure 5). These devices required extremely low optimal conditions for NO_2_ sensing with less than 20 μW to both bias and heat them, which was significantly lower than the 140 mW required for the external microheater. Furthermore, they have demonstrated the equivalence between thermal and room-temperature UV light-assisted responses of single SnO_2_ nanowire gas sensors [30,31] (Figure 6). For instance, the response of the sensors to 0.5 ppm NO_2_ at room temperature under UV light illumination was the absolute equivalent to that operating at 175 °C in dark conditions. The experimental results revealed that nearly identical responses, similar to thermally activated sensor surfaces, could be achieved by choosing the optimal illumination conditions.

Besides SnO_2_, In_2_O_3_, and ZnO nanowires or nanobelts, gas sensors based on single ZnO nanorods [32–35], single SnO_2_ nanotubes [36], single TiO_2_ and WO_2.72_ nanowires [37], and single NiO nanowires [38] have been reported. Liao *et al.* [39] have presented that the gas sensitivity of a single CeO_2_ (*E_g_* = 3.2 eV at 300 K) nanowire sensor to CO, H_2_, ethanol, gasoline, and H_2_S at room temperature could be significantly increased by incorporation of Pt nanoparticles on a CeO_2_ nanowire. In comparison to conventional metal oxide sensors, the Pt-sensitized single CeO_2_ nanowire sensor had an obvious advantage in selective detection of CO gas. However, the exact origin of the selectivity is still in question.

The above-mentioned gas sensors based on single 1D oxide nanostructure are summarized in Table 1. The target gases are mainly H_2_, CO, H_2_S, and NO_2_. The detection limits of the 1D oxide sensors are much lower than commercial thin film gas sensors based on metal oxide nanoparticles. The sensitivity of a few sensing methodologies has been improved greatly to allow the measurements of ambient level NO_x_ as those of the pre-existing high-end instrumental systems (e.g., chemoluminescent system [40–43]). Nonetheless, the performance of most sensors is still considerably lower in terms of sensitivity (e.g., by at least three or four orders of magnitude) than the top of the line instrumental set-ups (e.g., gas chromatographic methods for H_2_S [44–46] or spectrometric method for NH3 [47,48]). Although the single 1D nanostructure sensors can be much cheaper than the high instrumental systems, the practical application of the nanosensor technique might be possible after several key issues such as long-term stability, gas selectivity and low-temperature operation are resolved. These issues will be addressed in detail in Section 3 of this paper.

### 2.2. Multiple and Self-Assembled 1D Nanostructures

As reviewed in Section 2.1, gas sensors have been successfully fabricated using individual 1D nanostructures and demonstrated as a major platform for fundamental research. However, these devices are usually fabricated by pick-and-place process of a single 1D nanostructure, followed by formation of electrical contacts to the 1D nanostructures using expensive and time-consuming fabrication techniques such as electron-beam lithography. For 1D-nanostructure gas sensors to be viable for large-scale manufacturing, compatability of these devices with micro-fabrication tools such as conventional photo-lithography technique should be available.

**Figure 7.**
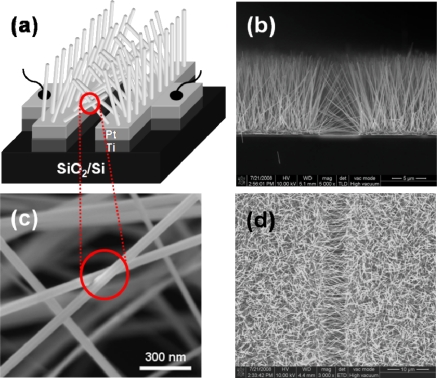
(a) The schematic illustration of ZnO-nanowire air bridges over the SiO_2_/Si substrate. (b) Side- and (d) top-view SEM images clearly show selective growth of ZnO nanowires on Ti/Pt electrode. (c) The junction between ZnO nanowires grown on both electrodes (Reprinted from reference [49] with permission from Elsevier).

Ahn *et al*. have developed novel on-chip fabrication of nanowire-based gas sensors, which is scalable and reproducible [49–51]. Figure 7(b) and (d) shows side- and top-view scanning electron microscope (SEM) images of ZnO nanowires grown on patterned electrodes. ZnO nanowires grown only on the patterned electrodes have many nanowire/nanowire junctions as seen in Figure 7(c). These junctions act as electrical conducting path for electrons. The device structure in this work is very simple and efficient compared with those adopted by previous researchers, because the electrical contacts to nanowires are self-assembled during the synthesis of nanowires. In other words, nanowire-based devices are usually fabricated either by tedious and time consuming processing steps such as electron-beam lithography to define the electrical contacts or by a series of processes involving synthesis, detachment of nanowires from the substrate by sonication, and dispersal of nanowires on another substrate with prefabricated electrodes.

Peng *et al*. [52] fabricated a gas sensor based on an assembly of porous silicon nanowires (SiNWs) by making electrical contacts on the top portion of as-prepared nanowires by thermal evaporation or other methods through a mask. The sensors made from the porous SiNWs assembly showed fast response and excellent reversibility to ppb-level NO concentration. The excellent sensing performance coupled with scalable synthesis of porous SiNWs could be a viable mass-production of sensor chips.

## 3. Critical Issues

The use of 1D metal oxide nanostuctures as gas sensors has potential advantages compared to conventional thin film devices due to the intrinsic properties of 1D nanostructures such as high surface-to-volume ratio and high crystallinity. However, the use of nanowires in real devices is still in a preliminary stage. Thus, how to integrate them with low-cost and high-yield mass production processes has become a major challenge for the future. Here, we discuss technological issues of long-term stability, selectivity, and room-temperature operation which act as bottleneck to the massive use of 1D metal oxide nanostructures in commercial gas sensors [53].

### 3.1. Long-Term Stability

Sysoev *et al.* [54] reported a comparative study of the long-term gas-sensing performance of gas sensors based on randomly oriented single crystal SnO_2_ nanowire mats and thin layers of pristine SnO_2_ nanoparticles. The sensing elements composed of percolating nanowires demonstrate excellent sensitivity and long-term stability toward traces of 2-propanol in air. The superior initial sensitivity of the nanoparticle layer deteriorated during the first month of the operation and approached to one observed steadily in the nanowire mats. The better stability of the nanowire mats sensors was explained in framework of reduced propensity of the single crystal nanowires to sinter under realworld operation conditions with respect to nanoparticle thin film. The stability of the percolating paths, analyte delivery and transduction mechanism in nanowire network sensing elements was defined at the microscopic level. Hernadez-Ramirez *et al.* [53] showed the advantages of individual nanowire-based sensors comparison to porous-film sensors that the absence of nooks and crannies in nanowire-based devices facilitates direct adsorption/desorption of gas molecules, improving the dynamic behavior of these prototypes to various gases. Especially, single-nanowire sensors have no contribution of necks and grain boundaries to the device operation, leading to good stability. However, the well-controlled manipulation and characterization of nanowires should be developed to produce stable and device-quality 1D nanostructure-based sensors.

### 3.2. Gas Selectivity

Ideal gas sensors are the ones that respond to only target gas molecules. Unfortunately, metal-oxide-based gas sensors respond in a similar way toward different oxidizing (or reducing) gas molecules, even though sensitivities might be different depending on the type of gas molecules. A variety of methods were proposed in metal-oxide gas sensors to improve the selectivity, such as particular bulk or surface doping, the application of gas specific prefilters, *etc.* [55–57].

**Figure 8.**
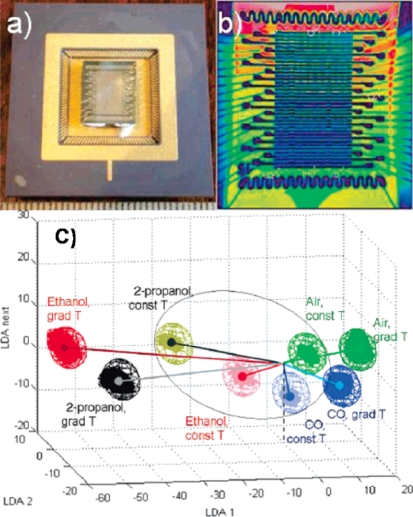
(a) KAMINA microarray chip with SnO_2_ nanowire sensing elements; (b) IR image of the chip under application of temperature gradient, 520 K (green area) −600 K (red area) along the electrode array; (c) LDA analysis of the conductivity patterns obtained with SnO_2_ nanowire-based gradient microarray at exposure to the sample gases (2–10 ppm concentration range). The classification spheres correspond to normal distribution of data at 0.9999 confidence level. The microarray operates under quasihomogeneous heating at 580 K (const T areas inside the ellipse with dimmed colors) and temperature gradient at 520–600 K (grad T areas with bright colors) (Reprinted from reference [59] with permission from American Chemical Society).

However, the identification of a specific target gas with a single sensing element is very challenging. Hagleitner *et al*. demonstrated single-chip gas sensor microsystem, which incorporated three different sensing mechanisms or mass-sensitive, capacitive and calorimetric ones and then selectively identified gas molecules using different responses of each gas molecule [58]. Similarly, an array of individual sensors with overlapping but different gas sensitivity can generate a unique pattern toward different specific target gas. The array of sensing elements can be constructed using different gas-sensing materials or different response of same sensing element as a function of operation temperature or surface/bulk doping. The unique pattern can be the fingerprint of the target gas. Using well-developed pattern recognition techniques, the response of the array of individual sensors can reliably discriminate the complex gas mixture and odors. The devices based on these sensing arrays are frequently referred to as electronic-nose because they mimic the principle of the mammalian olfactory system [59–61]. Sysoev *et al*. reported the practical gradient microarray electronic-nose (e-nose) with SnO_2_ nanowire gas-sensing elements, as seen in Figure 8 [59]. They constructed the array of SnO_2_ nanowire gas-sensors, which operate at different temperature zone from 520 K to 600 K. The device demonstrated an excellent performance as a gas sensor and e-nose system capable of promptly detecting and reliable discriminating between ethanol, 2-propanol and CO in air at a ppb level of concentration which cannot be detected by common olfactometer.

### 3.3. Room-Temperature Operation

Adsorption and desorption of gas molecules on the surface of metal oxides are both thermally-activated processes, which cause the response and recovery times to be usually very slow at room temperature. Thus, gas sensors based on 1D oxide nanostructures operate at high temperature (200–500 °C) to enhance the surface molecular adsorption/desorption kinetics and continuously clean the surface. Development of room-temperature gas sensors might have very important advantages such as low power consumption, simple system configuration, reduced explosion hazards, and longer device lifetime.

Desorption of gas molecules typically requires much higher activation energy than adsorption. Law *et al.* first demonstrated SnO_2_-nanoribbon-based gas sensor operating at room-temperature by desorbing attached NO_2_ gas molecules using ultraviolet (UV) irradiation [2]. UV-assisted desorption of NO_2_ was explained as follows: before UV illumination, oxygen species are adsorbed on the SnO_2_ nanoribbon surface, taking free electrons from the n-type SnO_2_ nanoribbon and forming a depletion region that extends into the thin nanoribbon. When the SnO_2_ nanoribbon is illuminated by UV light with wavelength shorter than the bandgap energy of SnO_2_, electron–hole pairs are generated. The positive holes discharge the negatively charged oxygen ions chemisorbed on the nanoribbon surface and eliminate the depletion region. Electrons produced at the same time increase the conductivity of the SnO_2_ nanoribbon. More recently, UV irradiation was reported to influence adsorption of gas molecules on the surface of SnO_2_ or ZnO nanostructures as well as desorption. Under illumination, photons partially desorb oxygen species from the surface, providing an increased number of adsorption sites available for gas molecules. Thus, the gas response of SnO_2_ or ZnO nanostructures with UV light irradiation was about 120 times higher than that without UV light irradiation [31,62].

Fan *et al*. reported highly sensitive room-temperature chemical sensors for detection of NO_2_ and NH_3_ based upon ZnO nanowire field-effect transistors [63]. The electric field applied over the back gate electrode modulates the carrier concentration, which in turn significantly affects adsorption and desorption behaviors of gas molecules or gas sensitivity. A strong negative field was utilized to refresh the sensors by an electrodesorption mechanism.

## 4. Summary

In this article, we review gas sensor application of 1D metal-oxide nanostructures with major emphases on the types of device structure and issues for realizing practical sensors. In the initial stage, gas sensors based on individual 1D nanostructure were successfully fabricated using electron-beam lithography and demonstrated excellent gas-sensing capability. As a result, the individual 1D-nanostructure gas sensors have been a platform technology for fundamental research. Recently, gas sensors with practical applicability were proposed, which were fabricated with an array of 1D nanostructures using scalable micro-fabrication tools such as conventional optical lithography. Some critical issues were addressed including long-term stability, gas selectivity, and room-temperature operation of the 1D-nanostructure-based metal-oxide gas sensors.

## Figures and Tables

**Table 1.** Properties of gas sensors based on single 1D oxide nanostructure.

**Target gas**	**Material**	**Sensor type**	**Detection limit (Temp.)**	**Sensitivity (Conc.)**	**Response time**	**Ref.**
NO_2_	SnO_2_ nanoribbon	Resistor	2 ppm (25 °C)	7 (100 ppm)	∼1 min	[2]
NO_2_	SnO_2_ nanowire	Resistor	<0.1 ppm (25 °C)	1 (10 ppm)	∼ 1 min	[31]
NO_2_	In_2_O_3_ nanowire	FET	0.5 ppm (25 °C)	10^6^ (100 ppm)	5 s	[15]
NO_2_	In_2_O_3_ nanowire	FET	0.02 ppm (25 °C)	0.8 (1 ppm)	15 min	[16]
H_2_	SnO_2_ nanobelt	FET	0.2% (25 °C)	0.17 (0.2%)	N/A	[4]
H_2_	SnO_2_ nanowire	FET	<1 ppm (200 °C)	4 (1 ppm)	∼50 s	[21]
H_2_	ZnO nanorod	Resistor	200 ppm (25 °C)	0.04 (200 ppm)	30−40 s	[35]
H_2_	VO_2_ nanowire	Resistor	N/A (50 °C)	1000 (100%)	∼10 min	[24]
H_2_	WO_2.72_ nanowire	Resistor	< 100 ppm (25 °C)	22 (1,000 ppm)	40 s	[37]
CO	SnO_2_ nanobelt	Resistor	5 ppm (400 °C)	7 (250 ppm)	30 s	[6]
CO	SnO_2_ nanowire	FET	100 ppm (25 °C)	15 (500 ppm)	∼10 min	[8]
CO	ZnO nanowire	Resistor	<50 ppm (275 °C)	3200 (400 ppm)	∼50 min	[10]
CO	NiO nanowire	Resistor	N/A (150 °C)	0.25 (800 ppm)	∼2 h	[38]
CO	CeO_2_ nanowire	Resistor	<10 ppm (25 °C)	2 (200 ppm)	∼10 s	[39]
H_2_S	SnO_2_ nanowire	Resistor	<1 ppm (150 °C)	6 × 10^6^ (50 ppm)	N/A	[9]
H_2_S	ZnO nanowire	Resistor	N/A (25 °C)	8 (300 ppm)	∼50 s	[13]
H_2_S	In_2_O_3_ nanowire	FET	1 ppm (25 °C)	1 (20 ppm)	48 s	[17]
Ethanol	SnO_2_ nanotube	Resistor	N/A (400 °C)	20 (7.8%)	∼80 s	[36]
O_2_	β-Ga_2_O_3_ nanowire	Resistor	<50 ppm (25 °C)	20 (50 ppm)	1 s	[26]
